# Suppression of low-frequency charge noise in superconducting resonators by surface spin desorption

**DOI:** 10.1038/s41467-018-03577-2

**Published:** 2018-03-20

**Authors:** S. E. de Graaf, L. Faoro, J. Burnett, A. A. Adamyan, A. Ya. Tzalenchuk, S. E. Kubatkin, T. Lindström, A. V. Danilov

**Affiliations:** 1 National Physical Laboratory, Hampton Road, Teddington, TW11 0LW UK; 2 Laboratoire de Physique Theorique et Hautes Energies, CNRS UMR 7589, Universites Paris 6 et 7, Place Jussieu, 75252 Paris, France; 3 L.D. Landau Institute for Theoretical Physics, Chernogolovka, 142432 Moscow Region, Russia; 4 Department of Microtechnology and Nanoscience, Chalmers University of Technology, SE-412 96 Göteborg, Sweden; 5 Royal Holloway, University of London, Egham, TW20 0EX UK

## Abstract

Noise and decoherence due to spurious two-level systems located at material interfaces are long-standing issues for solid-state quantum devices. Efforts to mitigate the effects of two-level systems have been hampered by a lack of knowledge about their chemical and physical nature. Here, by combining dielectric loss, frequency noise and on-chip electron spin resonance measurements in superconducting resonators, we demonstrate that desorption of surface spins is accompanied by an almost tenfold reduction in the charge-induced frequency noise in the resonators. These measurements provide experimental evidence that simultaneously reveals the chemical signatures of adsorbed magnetic moments and highlights their role in generating charge noise in solid-state quantum devices.

## Abstract (web summary)

The performance of solid-state quantum devices is often affected by low-frequency noise that appears to arise from the presence of two-level defects. Here the authors show that surface spins that cause magnetic noise are an origin of dielectric noise in superconducting resonators.

## Introduction

As the complexity of solid-state quantum circuits continues to increase, so do the challenges to both fabrication technology and materials science^1^. Improved device and systems engineering has led to material imperfections being a dominant source of noise and decoherence, and further improvements in material properties and a better understanding of the underlying materials physics are needed to make technologies such as large scale solid-state quantum computing feasible^1–3^. The enhanced sensitivity of superconducting qubits and resonators has revealed that materials once considered to be near-perfect crystals actually contain sufficient imperfections to behave as disordered systems. One unexpected consequence of the enhanced sensitivity to disorder of quantum devices was their ability to verify detailed predictions of the standard tunnelling model (STM)^4,5^. The STM, originally developed to model the low-temperature acoustical and electromagnetic properties of glasses, assumes the presence of a large ensemble of two-level systems (TLS) which can absorb energy via their electric dipole moments, leading to dissipation via subsequent phonon decay. TLS affect the performance of many different solid-state devices including superconducting resonators and qubits^3^, field-effect transistors^6^, single charge devices^7^ and ion traps^8^. Understanding and removing TLS are therefore important for a wide range of applications in solid-state physics, materials science, and chemistry.

While the origin of these TLS remains elusive, engineering advances have reduced TLS loss to a level where most remaining TLS are located at or in thin surface oxide layers^9–14^. In this regime the STM fails^15,16^. Remarkably, measurements of TLS-induced 1/*f* noise at low temperatures show an increasing noise ∝ *T*^−(1+*μ*)^, with *μ* ~ 0.3 found in both resonators^13,17,18^ and qubits^19^. This dependence, clearly different from the vanishing *T*^3^ dependence of the STM, is a signature of strong long-range TLS interactions. Furthermore, high quality (high-Q) resonators typically show a much weaker power dependence of the quality factor than what is predicted by the STM^18,20–22^. This prompted the development of a generalised tunnelling model (GTM)^16^ which takes into account of strong dipole–dipole interactions between TLS^23^, successfully capturing the observed physics.

Despite this success existing models do not give information about the chemical nature of surface TLS; something that is clearly needed for their mitigation. Directly studying the chemical nature of TLS using established surface analysis techniques remains extremely challenging. One reason is that the density of TLS is very small, <1% of surface sites, and likely comprised of very light elements^24^, weakly adsorbed molecules^25–27^ or electronic defect states^28^. These are easily introduced by exposing devices to ambient conditions^9,29^, inhibiting the use of many surface analysis techniques^30^. In constrast to charge TLS, magnetic dipoles as sources of flux noise originate from a bath of paramagnetic surface spins^9,10,31^, and can therefore be identified by on-chip electron spin resonance (cESR) techniques using sensitive tools derived from solid-state quantum technologies^10,29,32^. Identifying TLS that couple through their charge degree of freedom is much more challenging due to the lack of direct identification methods that can reveal chemical fingerprints.

In this work we show that changes observed in noise and loss measurements of superconducting resonators directly correlate with cESR data, which reveals important clues about the chemical and physical nature of surface TLS. By combining a range of different measurement techniques we show that superconducting resonators are versatile tools that can reveal a broad spectrum of noise mechanisms and decoherence mechanisms in materials used for quantum technologies. We further show that desorbing spins with a simple annealing treatment leads to a reduction of the frequency noise by almost an order of magnitude. This also allows us to directly identify the origin of the TLS responsible for noise as atomic scale electric dipoles; some of which are comprised of physisorbed atomic hydrogen^29^, while others are associated with free radicals. Our results suggest that these paramagnetic species not only cause a fluctuating magnetic environment^10^, but also are responsible for charge (dielectric) noise. Thus, not only cESR can be used to reveal the fingerprint of sources of flux noise, but the same technique will also be instrumental in identifying the origin of charge fluctuations in quantum devices and improving the coherence times of qubits.

## Results

### Experiments

We simultaneously measure the 1/*f* frequency noise and dielectric losses as a function of temperature and driving power (average photon number 〈*n*〉) of two NbN superconducting resonators (with frequencies *ν*_0_ = 4.6 GHz and 5.0 GHz)^33^ patterned on the same *c*-cut Al_2_O_3_ substrate.

The full high sensitivity cESR spectrum is subsequently obtained at *T* = 10 mK by measuring the quality factor of the resonator as a function of applied magnetic field and the zero field loss is subtracted to obtain the magnetic field induced loss $Q_{\mathrm{b}}^{ - 1}$^34^. We then anneal the device ex situ at moderate temperature (300 °C), a technique that has shown to remove some of the spins native to the surface of the device^29^. The same noise and loss measurement protocol is repeated in a second measurement and finally the cESR spectrum is measured again, confirming the successful removal of some of the spins. Throughout this paper we refer to these two consecutive measurements as ‘before’ and ‘after’ spin desorption respectively.

The frequency noise is measured in two resonators using a high precision dual Pound locking technique adapted from frequency metrology^35^ that continuously monitors the centre frequency of the resonators. Values for the dielectric loss tangent tan* δ*_0_ before and after annealing are extracted from quality factor measurements at high power, and from an independent measurement of the temperature-dependent frequency shift *ν*_0_(*T*) of the resonators we find the intrinsic loss tangent tan* δ*_i_. We note that care should be taken when comparing these two measures of loss, as they probe different quantities. For further details see Methods.

### Noise and ESR measurements

**Fig. 1:**
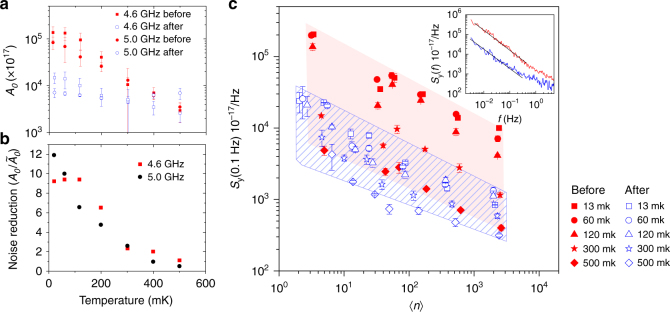
Reduction of noise due to surface spin desorption. **a** The extracted magnitude of the 1/*f* noise power *A*_0_ in the low power limit, obtained from the frequency noise power spectral density *S*_y_(〈*n*〉,* T*, *f*) = *A*_0_(〈*n*〉,*T*)/2*πf* as a function of temperature in two resonators before and after spin desorption. Error bars are 95% confidence bounds to fits of the power dependence of the noise data in **c**, including propagated errors. **b** The change in the magnitude of the noise before/after $= A_0/\tilde A_0$ vs. temperature. **a** and **b** are extracted from the full power and temperature dependence of the measured frequency noise power spectral density in **c**. **c** Frequency noise power spectral density $S_{\mathrm{y}}(f) = S_{\delta \nu }(f)/\nu _0^2$ at *f* = 0.1 Hz for the *ν*_0_ = 4.6 GHz resonator (see Supplementary Fig. 6 for 5.0 GHz resonator data). Red solid markers are before, and blue hollow markers are after spin desorption respectively. Shaded regions are to illustrate the range of noise powers covered by changes in temperature. The inset shows a typical 1/*f* noise power spectral density at 60 mK before ($\langle n\rangle \sim 200$) and after ($\langle n\rangle \sim 100$). Straight black lines are 1/*f*. Error bars indicate the standard deviation of the Allan deviation obtained for a series of timescales in the 1/*f* noise region (see Supplementary Note 9 for details)

The main result of this work is shown in Fig. 1. In summary, after annealing and desorption of surface spins we observe almost an order of magnitude reduction (on average 9.1 and 8.4 times for the two resonators respectively) in the frequency noise power spectral density for both measured resonators at the lowest temperatures.

**Fig. 2:**
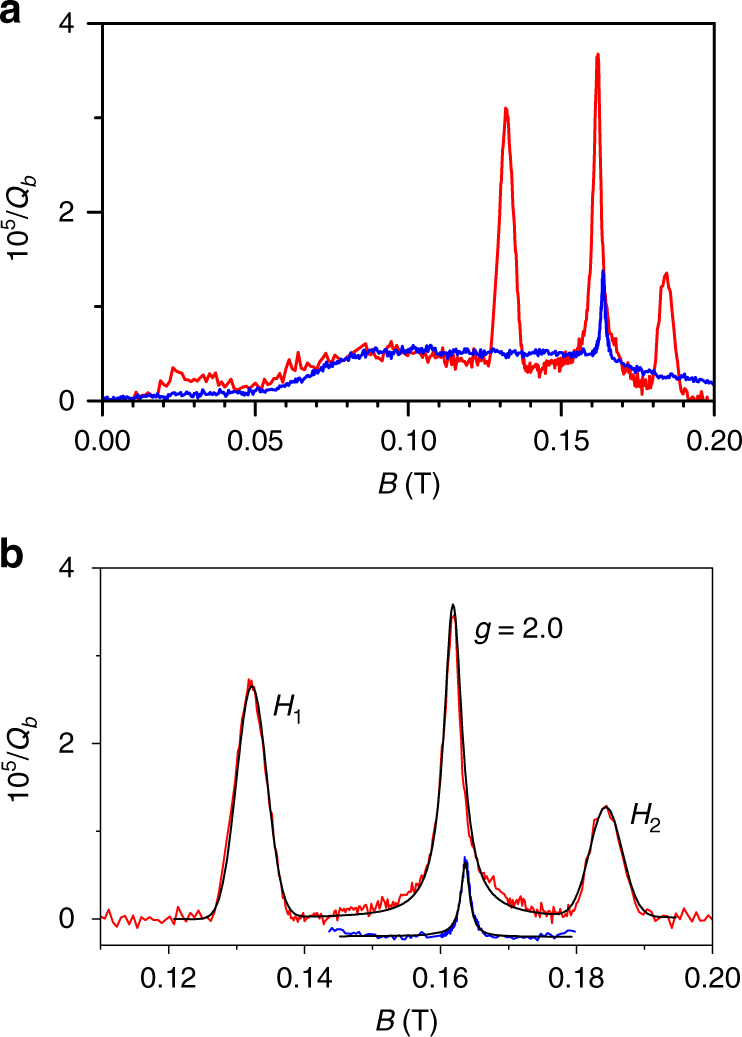
cESR spectrum. **a** The full cESR spectrum measured at 10 mK before (red) and after (blue) for the 4.6 GHz resonator, verifying that a large number of spins have been removed and **b** shows the same data zoomed in together with fit to theory (black lines) and the two hydrogen hyperfine peaks (*H*_1_ and *H*_2_) indicated together with the free electron peak *g* = 2.0 (see Supplementary Note 5–6 for further details). The wide background has been subtracted and curves have been offset for clarity

The reduction in noise is observed together with a reduction in number of surface spins. Figure 2 displays the cESR spectrum measured in situ after collecting all the noise data, before and after annealing. The measured cESR spectrum reveals the presence of atomic hydrogen on the Al_2_O_3_ surface originating from water dissociation^36^ and electronic charge states (with a *g*-factor of 2.0), likely due to absorption of oxygen radicals on the surface in accordance with previous findings^9,29^. An initial density of *n*_H_ = 2 × 10^17^ m^−2^ hydrogen spins is completely removed and we extract a reduction in spin density due to the central peak from *n*_e_ = 0.91 × 10^17^ m^−2^ to $\tilde n_{\mathrm{e}} = 0.17 \times 10^{17}$ m^−2^, a factor of 5.3. The estimated uncertainty for these absolute numbers is about 10%. The wide background plateau, expected to be the result of a similar number of spins, remained unchanged.

Intriguingly, in contrast to the tenfold reduction in noise, we find that the intrinsic loss tangent tan* δ*_i_ is only reduced by 30% after surface spin desorption. For each resonator we also measured the power and temperature dependence of the quality factor, from which we also see only a very small change in the loss (see Table 1 for exact values).

**Table 1:** Extracted parameters from cESR and noise/loss measurements

Quantity	Unit	Before	After	Note
Spin density^a^	10^17^ m^−2^	0.91	0.17	*g* = 2
		2.0	0	H
*F* tan *δ*_i_	×10^−6^	10.6 ± 0.15	7.44 ± 0.13	4.6 GHz
		10.4 ± 0.27	7.69 ± 0.12	5.0 GHz
*P*_*γ*_*F* tan* δ*_i_	×10^−6^	4.2 ± 0.24	4.9 ± 0.1	4.6 GHz
		5.4 ± 0.6	6.5 ± 0.6	5.0 GHz
*P*_*γ*_		0.39 ± 0.02	0.66 ± 0.02	4.6 GHz
		0.52 ± 0.06	0.84 ± 0.08	5.0 GHz
*α*		0.20 ± 0.024	0.18 ± 0.037	4.6 GHz
		0.27 ± 0.02	0.22 ± 0.038	5.0 GHz
2*μ*		0.64 ± 0.50	0.43 ± 0.21	4.6 GHz
*A*_0_/2*π*	10^−17^	2.2 ± 0.3 × 10^4^	2.4 ± 0.4 × 10^3^	4.6 GHz
		1.2 ± 0.4 × 10^4^	1.1 ± 0.3 × 10^3^	5.0 GHz

For a detailed description of each parameter see refs. ^16,18,29^ and the Supplementary Notes 1–11.Where indicated, deviations are 95% confidence bounds or propagated errors thereof from fitting

^a^ For the 4.6 GHz resonator

## Discussion

**Fig. 3:**
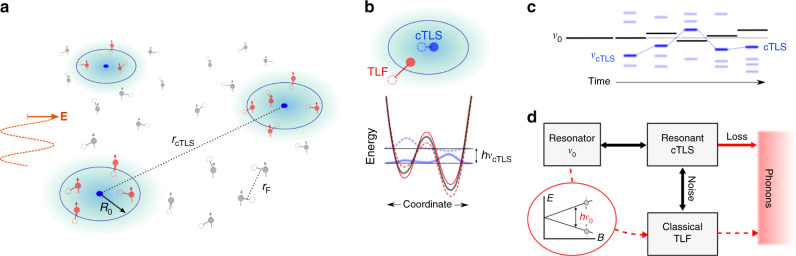
Mechanism of frequency noise and loss in high-Q superconducting resonators. **a** A smaller number of coherent two-level systems (cTLS) (blue) on average separated by a distance $r_{{\mathrm{c}}\mathrm{TLS}} \sim 1$ μm couple to the oscillating electric field component *E* of the resonator. Classical thermally activated two-level fluctuators (TLFs) (red) in *R*_0_ ~ 60 nm proximity of the cTLS generate noise, while other thermally activated TLFs (grey) contribute to the cTLS line width and the total density of TLS detected in cESR measurements. Typical distances between thermally activated TLF (at *T* ~ 60 mK) are *r*_F_ ~ 100 nm. **b** TLF inside the interaction volume of the cTLS modify the tunnelling potential of the cTLS, resulting in the cTLS energy drift. **c** The resonantly coupled cTLS have energy level splittings near the resonance frequency *ν*_0_. This splitting fluctuates in time, perturbing the resonator frequency via its coupling to the electric dipole associated with the cTLS. **d** The conceptual representation of the generalised tunnelling model where noise and loss channels are indicated (see text). The cESR measurement enables identification of TLFs via the new dissipation channel, indicated by dashed lines, arising when the spins are in resonance with the microwave field

This small reduction in loss but large reduction in noise can be explained within the framework of strongly interacting TLS and the GTM, which naturally partitions the TLS as two distinct entities, one predominantly responsible for loss and one for noise. The microscopic picture is the following. Associated with each TLS there is a fluctuating dipole *d*_0_ that couples to the applied microwave electric field *E* from the resonator. Among the TLS we can distinguish between coherent (quantum) electric dipoles (cTLS) coherent two-level systems that are characterised by fast transitions between their states and relatively small decoherence rates, and slow classical fluctuators (from now on referred to as TLF) that are characterised by decoherence times shorter than the typical time between the transitions. The picture is sketched in Fig. 3a with typical distances between thermally activated (excited) cTLS and TLF as inferred from our measurements.

At low temperatures, slow fluctuators weakly coupled to cTLS mainly contribute to the dephasing of the high energy cTLS and are responsible for their line-width *Γ*_2_^16,37^. Slow fluctuators that are located close to the cTLS, and therefore are strongly coupled, shift the cTLS energy by an amount larger than *Γ*_2_. These fluctuators create highly non-Gaussian noise that cannot be regarded as a contribution to the line width. For resonant cTLS, having an energy splitting *E* ≈ *ħν*_0_, the interaction with a few strongly coupled TLF translates to the energy of the cTLS drifting in time, as illustrated in Fig. 3b, c; it is this drift that ultimately generates 1/*f* noise in the resonator^16^. The same drift has another important consequence: we cannot rely on measurements of the loss (*Q*_i_) at low powers to probe the average density of cTLS. Indeed, at low power the loss is determined by the imaginary part of the response which is strongly peaked around *ν*_0_^16^, making *Q*_i_ dependent on the cTLS dynamics, as well as the local density of states around *ν*_0_. The average density of cTLS can instead be reliably determined by measuring *ν*_0_(*T*) which is given by the real part of the response. The *ν*_0_(*T*) dependence probes cTLS in a broad range of energies, making this effect insensitive to cTLS dynamics.

Within the framework of the GTM our experimental findings of a small reduction in loss and a dramatic reduction in noise imply that desorption of surface spins did not affect the density of cTLS, instead the surface spins can be attributed to the TLF.

The conceptual picture of these two separate TLS communities is further supported by two additional experimental findings: for a homogeneous bath of non-interacting TLS (STM) we expect $Q_{\mathrm{i}}(\langle n\rangle ) \sim \langle n\rangle ^\alpha$ with *α* = 0.5. The observed dependence is much weaker: a fit to a power law returns *α* ≈ 0.2 for both resonators before and after desorption (see Supplementary Note 4). This is a signature of interacting TLS, from which we expect a much weaker logarithmic dependence of the microwave absorption on stored energy in the resonator^22^

1 $$\frac{1}{{Q_{\mathrm{i}}(\langle n\rangle )}} = P_\gamma F\,{\mathrm{tan}}\,\delta _{\mathrm{i}}\,{\mathrm{ln}}\left( {C\sqrt {\frac{{|n_c|}}{{|\langle n\rangle |}}} + c_0} \right).$$

**Fig. 4:**
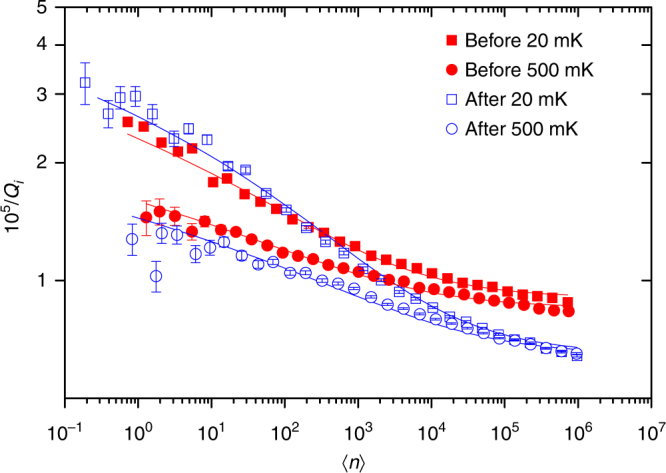
Resonator quality factor. Inverse internal quality factor as a function of number of photons in the 4.6 GHz resonator. Solid lines are fits to the logarithmic power dependence of eq. (1) for $\langle n\rangle \sim 50$ (in the low power saturation regime eq. (1) is not valid) and the fitted curve is extended to lower powers. Extracted values are reported in Table 1. Error bars are 95% confidence bounds from fits to the measured *S*_21_ line shape

Here *C* is a constant, *c*_0_ accounts for power-independent losses, *F* is a geometric filling factor and *P*_*γ*_ is a normalisation factor that depends on the spectral density of TLF switching rates. In Fig. 4 we show that our data fits very well to this logarithmic power dependence.

Interestingly, we find that *P*_*γ*_ increases after spins were removed. This implies that the remaining slow fluctuators have a narrower range of switching rates and are likely different in nature than the spins that were desorbed. We also note that the observed reduction in tan *δ*_i_ accompanied by an increase in the low power 1/*Q*_i_ is an expected outcome of cTLS dynamics.

Independently, another important indication of the applicability of our model is given by the analysis of the temperature dependence of the 1/*f* noise spectrum. The interaction gives a vanishing density of states for cTLS at low energies, *P*(*E*) ∝ *E*^*μ*^ with 0 < *μ* < 1, and this results in a scaling of the noise spectrum with temperature *S*_y_(*T*) ∝ *T*^−(1+2*μ*)^ (for *T* < *hν*_0_/*k*_B_). In agreement with previous studies^13,17–19^ we find *μ* ≈ 0.3 (see Supplementary Note 10), both before and after spin desorption. This is further evidence that desorption only affects the number of slow TLF present on the sample.

We now combine all available data to produce a qualitative picture (as sketched in Fig. 3) of the microscopic properties of the cTLS and TLF, by taking the GTM beyond the original assumptions of identical densities and dipole moments of cTLS and TLF^16,18^. The details of this theory and analysis can be found in the Supplementary Note 1, here we only summarise the results.

Assuming the dipole moment for resonant cTLS to be on the atomic scale, $d_0 = 1\,{\mathrm{e{\AA}}} \sim 5{\mathrm{D}}$ (i.e., similar to what was previously deduced from spectroscopy measurements^38,39^), we arrive at dipole–dipole interaction strength *U*_0_ ≈ 15 Knm^3^. Before spin desorption, we find from the intrinsic loss tangent the cTLS line-width $\Gamma_2 \sim 20 \, {\mathrm{MHz}}$ at *T* = 60 mK (see Supplementary Note 1), which translates into the density of resonant cTLS *ρ*_c*TLS*_ ≈ 15 GHz^−1^ μm^−2^, in close agreement with the density of TLS found in qubit tunnel junctions^37,38^. This means that resonant cTLS are located at a typical distance $r_{{\mathrm{c}}\mathrm{TLS}} \sim 1$ μm from each other.

Next, the measured magnitude of the 1/*f* noise, *A*_0_, can be related to the density of thermally activated (fluctuating) TLF and their dipole moment *d*_F_. We find (*d*_F_/*d*_0_)*ρ*_F_ ≈ 5 ± 4 × 10^−3^ nm^−2^. The thermally activated TLF constitutes a fraction *T*/*W* of the total number of TLF, where *W* is the bandwidth of the distribution of TLF energy level splittings. For weakly absorbed spins it is reasonable to expect that *W* ~ 100 K, limited by the observed desorption energy. From the total spin density measured by cESR we have *n*_e_ + *n*_H_ ≈ 3 × 10^−1^ nm^−2^.

Combining these estimates, assuming all the TLFs are the observed spins, we have for the density of thermally activated TLF $\rho _{\mathrm{F}} = (n_{\mathrm{e}} + n_{\mathrm{H}})(T/W) \sim 2 \times 10^{ - 4}\,{\mathrm{nm}}^{ - 2}$ (i.e., thermally activated TLFs are separated by an average distance $r_{\mathrm{F}} \sim 100\,{\mathrm{nm}}$) and $d_{\mathrm{F}}/d_0 \sim 30 \pm 25$. The large uncertainty in *d*_F_/*d*_0_ stems from its strong dependence on the filling factor (∝ *F*^3^) and the volume where the TLF are situated, which both cannot be accurately estimated. However, the message of this order of magnitude estimation is that the assumption that all TLF are the observed spins is indeed plausible. Furthermore, the dipole moment of a surface TLF is likely larger compared to that of TLS in the bulk, as would be expected since the physisorbed and easily desorbed spins are likely to move larger distances.

After spin desorption the magnitude of the 1/*f* noise decreases by a factor ~10, the loss is only reduced by ~30% and the normalisation constant *P*_*γ*_ increases ~65% due to lower TLF switching rates. From this we can finally find a corresponding change in the density of TLF before and after spin desorption

2 $$\frac{{\rho _{\mathrm{F}}(T)}}{{\tilde \rho _{\mathrm{F}}(T)}} = \frac{{A_0\tilde P_\gamma }}{{\tilde A_0P_\gamma }} = 16\, {\mathrm{and}}\, 18,$$

for the two resonators. Here we denote quantities for the ‘after’ measurement by the tilde symbol. These values correlate remarkably well with the change in the total number of spins in the three cESR peaks $(n_{\mathrm{e}} + n_{\mathrm{H}})/\tilde n_{\mathrm{e}} = 17 \pm 2$ (4.6 GHz resonator), and again indicates that spins contribute to the frequency noise in our high-Q superconducting resonators and take on roles as slow (mobile^36^) fluctuators.

Based on the experimental evidence from the loss, noise and cESR spectrum, all obtained on the same device, we have found that surface spins that are known to give rise to magnetic noise in quantum circuits^10,29,31^ are also responsible for the low frequency charge noise of the resonator. These spins, remarkably present in densities also inferred to be responsible for flux noise in SQUIDs and qubits^31^, take on roles as slow classical fluctuators that cause an energy drift of resonant coherent TLS. Removing a majority of these spins gives an almost tenfold reduction in charge (dielectric) noise.

In our device the observed surface spins constitute weakly physisorbed atomic hydrogen together with free radicals (*g* = 2). We note that the nature of the *g* = 2 spins is still not entirely clear. A large portion can be associated with surface adsorbates, likely oxygen radicals^9,29^, or other light molecular adsorbents^25,27^. The remaining fraction of free radicals may be a result of insufficient annealing or they may be of a different chemical or physical origin with much higher desorption barriers. Another possibility is that the remaining more robust localised charges and cTLS are intrinsic to the Al_2_O_3_ surface itself^28,36^, more resembling bulk defects^40^. The remarkable stability of the spin populations in prolonged exposure to atmospheric conditions likely originates from the initial surface hydroxylation inhibiting further generation of spin active surface species^29^. Nevertheless, our approach reveals that observed magnetic dipoles, with their fingerprint revealed through surface analysis using in situ on-chip ESR, couple via the electric field degree of freedom and give rise to charge noise. Our combination of state-of-the-art measurement techniques reveals an until now unknown link between classical (charge) fluctuators and spins— primarily suspected to be the cause of flux noise in for example SQUIDs.

Similar physics is expected for a wide range of oxide surfaces relevant for quantum technologies. The importance of magnetic moments has previously been widely overlooked in resonators since electric dipoles have been considered the dominating mechanism for charge noise. Our results instead indicate that while having a small influence on power loss, these spins (and their associated electric dipoles) constitute a major source of noise and dephasing in modern high coherence solid-state devices by their proximity to coherently coupled resonant cTLS, and our results hint at a connection between the similar densities found for sources of flux^31^ and charge^7,41^ noise in quantum circuits.

## Methods

### Sample preparation

Sapphire substrates were annealed in situ at high temperature, 800 °C, for 20 minutes prior to deposition of 2 nm NbN. After cooling down to 20 °C, an additional 140 nm NbN was sputtered. Resonators were patterned using electron beam lithography (UV60 resist, MF-CD-26 developer, DI water rinse) and subsequent reactive ion etching in a NF_3_ plasma. Resist was removed in 1165 remover followed by oxygen plasma treatment. Resonator designs were identical to those reported in ref. ^33^. After the first round of noise measurement the same sample was warmed up, shipped from UK to Sweden, and heated in vacuum (≈10^−8^ mBar) to ~300 °C for 15 minutes to desorb surface spins, then shipped back to the UK sealed in a vacuum-sealed plastic bag, and mounted in the same cryostat with the same noise measurement setup ~72 h later. Remarkably, the detrimental surface spins are not re-introduced even after this time and after several hours of exposure to ambient conditions.

### Measurement setup

We used a cryogen-free dilution refrigerator with a base temperature of 10 mK and a three-axis superconducting vector magnet for noise and cESR measurements. The cryostat was equipped with heavily attenuated coaxial lines, cryogenic isolators and a low noise high electron mobility transistor (HEMT) amplifier with a noise temperature of ~4 K. All noise measurements were performed with the leads to the vector magnet completely disconnected. Only after completion of noise measurements the magnet was connected to measure the cESR spectrum. The plane of the superconductor thin film was found to high precision (<0.1°) by applying a small field and carefully tilting the angle of the applied field while finding the maximum of the resonance frequency of the resonators. cESR measurements were performed by sweeping the magnetic field and measuring the characteristics of the resonators using a vector network analyser. Noise measurements were performed using a Pound locking technique^35^ that tracks the resonance frequency (and its fluctuations) in real time. For a detailed explanation of the technique, see Supplementary Note 7.

### Data availability

The data that support the findings of this study are available from the corresponding author upon reasonable request.

## Electronic supplementary material

Supplementary Information(PDF 595 kb)
