# Supplementary material for: Suppression of low-frequency charge noise in superconducting resonators by surface spin desorption
Source: Nat Commun. 2018 Mar 20;9:1143. doi: 10.1038/s41467-018-03577-2 (PMC5861058; doi:10.1038/s41467-018-03577-2)
Supplement: Supplementary file 1 — Supplementary Information(PDF 595 kb) [file 41467_2018_3577_MOESM1_ESM.pdf]

Supplementary Information

Suppression of low-frequency charge noise in superconducting resonators by surface spin desorption

S. E. de Graaf et al.

SUPPLEMENTARY NOTE 1. MODEL FOR INTERACTING TLS

Our experiments on noise and loss indicate that interactions between TLS are important. They also demonstrate that while the spin desorption procedure significantly affects the magnitude of the noise it has only a minor effect on the intrinsic loss tangent. In this section we discuss the full microscopic model of interacting TLS and their physical origin that follows from the data.

The fact that surface spin removal has a small effect on the loss tangent implies that the spins do not contribute significantly to the loss at high frequency and thus are not a part of the resonant cTLS ensemble. However, the reduced noise implies that the spins are a significant host of the bath of slow fluctuating dipoles (TLF). The cESR spectrum also indicates that the desorbed spins (both H and free electron states) can be highly mobile and can tunnel a long distance, i.e. they can easily serve as the dominant fraction of slow fluctuating dipoles.

Coherent cTLS are associated with fluctuating dipoles d_0 and are described by the Hamiltonian $H_{\rm TLS} = \frac{\Delta}{2}\sigma^z + \frac{\Delta_0}{2}\sigma^x$ characterized by an asymmetry Δ, tunneling matrix element Δ_0, and $\sigma^a, a = x, y, z$ are the Pauli matrices. In the rotated basis, the Hamiltonian is simply $H_{\rm TLS} = ES^z$, where $E = \sqrt{\Delta^2 + \Delta_0^2}$ is the TLS energy splitting and $S^z = \dfrac{1}{2}(\cos\theta\sigma^z + \sin\theta\sigma^x)$ with $\tan\theta = \Delta_0/\Delta$. The interaction strength is set by the dipole-dipole interaction scale $U_0 = d_0^2/\varepsilon_{\rm h}$, where $\varepsilon_{\rm h} = 10$ is the dielectric constant of the host medium. As a consequence of this interaction, the TLS density at low energies is $P_{\rm TLS}(E, \sin\theta) = \frac{P_0}{\cos\theta\sin\theta}\left(\frac{E}{E_{\max}}\right)^{\mu}$, where $\mu < 1$ is a small positive parameter. Among coherent TLS we distinguish high, $E \gg k_{\rm B}T$, and low $E \leq k_{\rm B}T$ (thermally activated) energy TLS. In addition, some TLS can be (near) resonant with the resonator, $E \sim h\nu_0$.

The slow fluctuators are represented by classical fluctuating dipoles with moment $d_{\rm F}$, characterized by switching rates γ with a probability distribution $P_{\rm F}(E, \gamma) = P_0^{\rm F}/\gamma$ and $\gamma_{\min} \ll \gamma \ll \gamma_{\max}$. Such a distribution for the switching rates appears naturally for thermally activated tunnelling.

The loss in a high quality resonator is caused by fluctuating dipoles with energies close to the resonator frequency ν_0. In the regime of low temperature, $k_{\rm B}T \ll h\nu_0$, the resonant cTLS have a small dephasing width due to their interaction with thermally activated TLS and TLF. This width is given by [1]

$$\Gamma_2 = \ln\left(\frac{\Gamma_1^{\max}}{\Gamma_1^{\min}}\right)\chi\frac{T^{1+\mu}}{\nu_0^{\mu}}, \tag{1}$$

where $\chi = P_0U_0\left(\frac{\nu_0}{E_{\max}}\right)^{\mu} \approx \tan\delta_{\rm i}$ is a dimensionless parameter, obtained directly from loss tangent measurements, that controls the effect of the interaction on the resonant cTLS. $\Gamma_1^{\max}$ and $\Gamma_1^{\min}$ are the minimum and maximum relaxation rates of these cTLS respectively. Direct measurements give $\Gamma_1^{\max} \approx 10^4$ s^{-1} for the thermally activated cTLS at $T \approx 35$ mK[2]. The precise value of $\Gamma_1^{\min}$ for thermally activated cTLS is not known. However, the electrical noise data shows that $1/f$ noise generated by these cTLS extends to very low frequencies $f \leq 1$ mHz beyond which the dependence changes. This implies that $\Gamma_1^{\min} \approx 10^{-3}$ s^{-1}, such that $\ln\left(\Gamma_1^{\max}/\Gamma_1^{\min}\right) \approx 20$.

The total number of resonant cTLS in a volume $V_{\rm h}$ ($= 2.4$ and $2.2 \cdot 10^{-16}$ m^3 for the two resonators respectively) of host material can then be estimated from the measured loss tangent as $\mathcal{N}_{\rm res} = \frac{\chi}{U_0}V_{\rm h}\Gamma_2 \sim 10^4$; their average distance is $r_{\rm cTLS} \sim (\chi\Gamma_2/U_0)^{-1/3}$ in bulk material and $r_{\rm cTLS} \sim (d\chi\Gamma_2/U_0)^{-1/2}$ in a thin film of thickness $d \ll r_{\rm cTLS}$.

The noise in the resonator is due to the slow TLF that interact strongly with these resonant cTLS and create highly non-Gaussian noise, that cannot be regarded as a contribution to Γ_2. These TLF are located at distance $r < R_0$, where $R_0^3 = \frac{d_{\rm F}}{d_0}\frac{U_0}{\Gamma_2}$. Their switchings bring the cTLS in and out of resonance with the resonator leading to $1/f$ frequency noise. The number of thermally activated TLF strongly coupled to a resonant cTLS is $\mathcal{N}_{\rm F}(T) = P_0^{\rm F}\frac{4\pi}{3}R_0^3T$ and their average distance is $r_{\rm F} \sim (P_0^{\rm F}T)^{-1/3}$. If the total number of such fluctuators, $\mathcal{N}_{\rm F}^{\rm tot}(T) = \mathcal{N}_{\rm res}\mathcal{N}_{\rm F}(T) \gg 1$, the frequency noise spectrum of the resonator can be expressed as a superposition of Lorenztians generated by the switching of the TLF strongly coupled to the resonant cTLS.

In the limit of weak electric field $\mathbf{E}$ we find that the noise is given by[1]

$$\frac{S_{\delta\nu}}{\nu_0^2} = \frac{8}{15}\langle d_0^4\rangle\frac{\chi}{U_0\Gamma_2}\mathcal{F}(\mathbf{E})\mathcal{N}_{\rm F}(T)\int_{\gamma_{\min}}^{\gamma_{\max}}\frac{\gamma P(\gamma)}{\gamma^2+\omega^2}d\gamma. \tag{2}$$

Here $P(\gamma) = P_\gamma/\gamma$ is the normalized distribution function of slow fluctuators with $P_\gamma = \ln^{-1}[\gamma_{\max}/\gamma_{\min}]$ and $\mathcal{F}(\mathbf{E}) = \dfrac{\int_{V_{\rm h}}|\mathbf{E}|^4dV}{4(\int_V\varepsilon_0|\mathbf{E}|^2dV)^2} \approx \dfrac{F^2}{\varepsilon_{\rm h}^2V_{\rm h}}$ where we introduced the filling factor $F = \dfrac{\int_{V_{\rm h}}\varepsilon_{\rm h}|\mathbf{E}|^2dV}{2\int_V\varepsilon_0|\mathbf{E}|^2dV} \sim 0.01 - 0.02$ [3] which accounts for the fact that the TLS host material volume $V_{\rm h}$ may only partially fill the resonator mode volume V. We note that the uncertainty in accurately determining F gives a large range for the possible dipole moment ratio $d_{\rm F}/d_0$. The ranges given for the quantities in the discussion on $d_{\rm F}$ in the main manuscript are the values obtained for the estimated range of the filling factor. Notice that in this limit the noise spectrum scales with temperature as $\propto T^{-(1+2\mu)}$. Eq. (2) gives the $1/f$ noise spectrum

$$\frac{S_{\delta\nu}}{\nu_0^2} = \frac{A_0}{2\pi f}. \tag{3}$$

The coefficient A_0 can be expressed through the total

number of thermally activated fluctuators $\mathcal{N}_{\rm F}^{\rm tot}(T)$ as

$$A_0 \approx \pi \frac{d_{\rm F}}{d_0} \mathcal{N}_{\rm F}^{\rm tot}(T) \left[F^2 P_\gamma\right] \left(\frac{U_0}{\Gamma_2 V_{\rm h}}\right)^2 . \quad (4)$$

SUPPLEMENTARY NOTE 2. DEVICE DETAILS

Here we briefly outline the main properties of the superconducting resonators used in this study. The geometry of our resonator is that of a folded $\lambda/2$ resonator, optimised for increased coupling to surface spins and high resilience to magnetic field; these characteristics make this type of resonator ideal for cESR studies. A detailed description of the geometry can be found in Ref. [4]. Here we show the electric field distribution in a segment of the resonator in Supplementary Figure 1. It can be seen that the microwave electric field is strongly localised to the 2 μm wide gap between the two electrodes in our 2-prong design. The field distribution and filling factor of the TLS host volume $F \approx 0.02$ was obtained using COMSOL finite element calculations. To evaluate the filling factor we integrate the field strength over the whole surface not covered by metallic electrodes. We note that the electric field strength varies by approximately one order of magnitude in the gap between the electrodes where the coupled spins are located, with an increased field only in close vicinity of the metallic electrodes. The magnetic field distribution (used for evaluation of spin density) is discussed in detail in Ref. [5] and in the supplemental material of Ref. [6].

SUPPLEMENTARY NOTE 3. NUMBER OF PHOTONS

Supplementary Figure 2a shows the number of photons in the 4.6 GHz resonator vs internal loss ($Q_{\rm i}^{-1}$) for the two measurements at two temperatures. Each measurement was made in the same sample cell using the same microwave setup, and the initial assumption is that in the two separate measurements the attenuation in the cryostat was the same. Each data point corresponds to a 2 dB increment in the applied power, both datasets starting at the same low applied power. Therefore, the range of microwave powers applied to the sample is expected to be the same across both measurements. This is further validated by the measurement of white noise levels that are the same (within a factor 2). The white noise level in these measurements is dominated by the microwave power incident on the cryogenic amplifier.

The number of photons within the resonator scales with the loaded quality factor, and therefore also with the internal quality factor. As discussed in the main text, the spin desorption leads to an increase in $Q_{\rm i}$, meaning that for the same applied microwave power, the number of photons in the resonator is different between the "before" and "after" measurements. Importantly, the noise scales with both the number of photons within the resonator and with $Q_{\rm i}$. As is customary in the literature, we calibrate the applied power such that we compare noise for the same number of photons within the resonator.

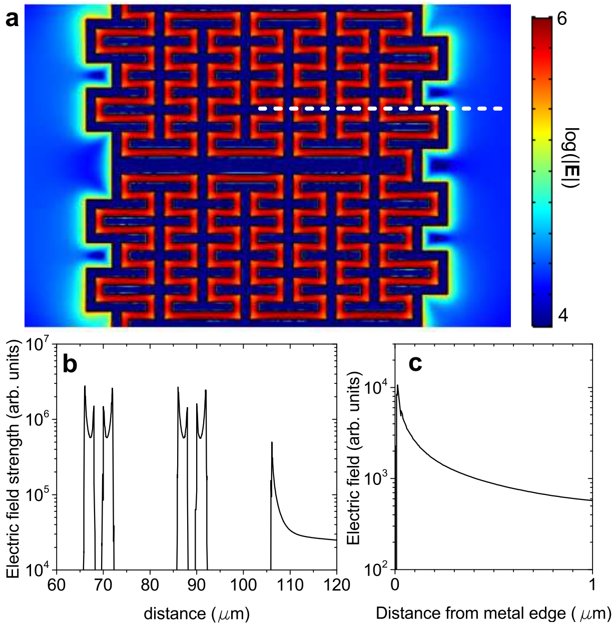

Supplementary Figure 1. Electric field distribution. a) Electric field distribution evaluated at the substrate-air interface in a segment of our resonator. b) cross section showing the electric field distribution along the dashed line in a). c) close-up on the electric field from the edge of one of the numerous metallic edges.

SUPPLEMENTARY NOTE 4. POWER DEPENDENCE OF $Q_{\rm i}$

For consistency we here also provide the analysis of the quality factor data within the framework of the STM. Here we expect at strong fields $\langle n\rangle \gg n_c$

$$Q_{\rm i}^{-1} = \frac{F \tan\delta_0}{(1+\langle n\rangle/n_c)^\alpha} + Q_{\rm i,0}^{-1}, \quad (5)$$

where the constant $Q_{\rm i,0}$ accounts for power-independent loss and n_c is a critical photon number for saturation, $\alpha = 0.5$ (for any non-interacting TLS) and F is the filling factor of the TLS hosting medium in the resonator. By fitting the measured $Q_{\rm i}(\langle n\rangle)$ to this power law we find $\alpha \sim 0.2$ both before and after spin desorption. Typical fits can be seen in Supplementary Figure 2. We note that electric field non-uniformity may lead to different critical photon numbers in the STM [7], however, at high fields $\alpha = 0.5$ is always recovered for non-interacting TLS. Here we also emphasise that care should be taken when comparing the quantity $F\tan\delta_0$ with the loss tangent (see below) extracted from temperature dependent frequency shifts of the resonator, $F\tan\delta_{\rm i}$, in the low power limit.

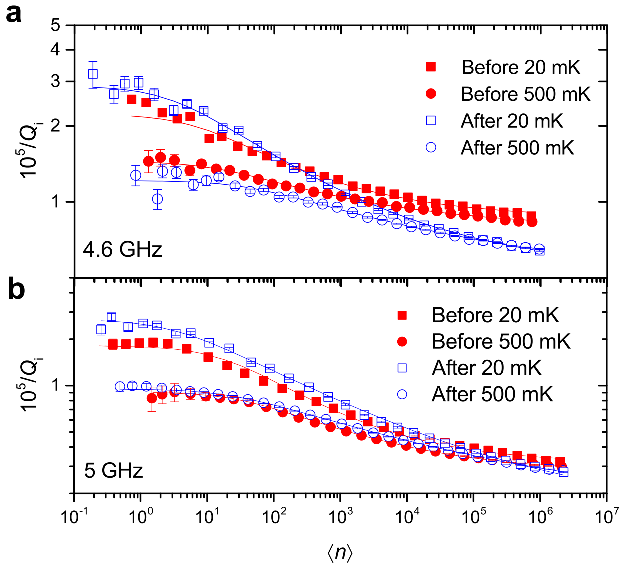

Supplementary Figure 2. Inverse internal quality factor as a function of number of photons in the resonator for two extreme temperatures covering those used in all other measurements. Each data point is an increment in applied power by 2 dB, starting at the same low applied power. Fits are to equation (5). Error bars are 95% confidence bounds from fits to the measured S_{21} line shape. Some of the data is the same as in Figure 4 in the main manuscript.

This as the former probes the cTLS density of states in a narrow window around ν_0 and the latter is a measure of the average cTLS density of states.

SUPPLEMENTARY NOTE 5. CESR-SPECTRUM AND SPIN DENSITY

The cESR-spectrum in Figure 2 in the main manuscript is obtained by measuring the transmitted microwave signal, S_{21}, around resonance as a function of applied magnetic field using a vector network analyser. All noise measurements were performed first, making sure the resonator was not poisoned by vortices. Once noise measurements were completed, the superconducting magnet leads were connected and a magnetic field applied in the plane of the superconducting film. The measured microwave transmission was fitted to [8]

$$S_{21} = 1 - \frac{(1 - S_{21,\mathrm{min}})e^{i\varphi}}{1 + 2iQ\frac{\nu_0 - f}{\nu_0}}, \qquad (6)$$

to extract the internal quality factor $Q_\mathrm{i} = Q/S_{21,\mathrm{min}}$. The parameter φ accounts for the asymmetry in the resonance line-shape accounting for possible impedance mismatch. The spin-induced loss is then calculated as

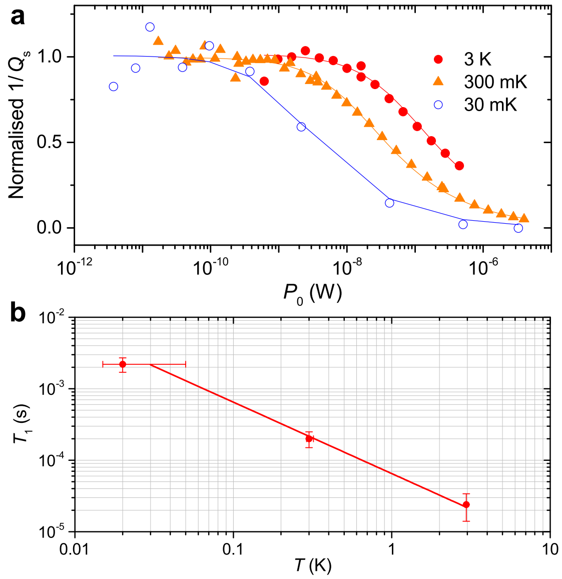

Supplementary Figure 3. Spin relaxation times. a) CW power saturation measurements at three different temperatures showing the normalised inverse dissipation into the spin system as a function of circulating power in the 4.6 GHz resonator after annealing. b) the extracted (from fits) relaxation time T_1 as a function of temperature for the $g = 2$ peak. Solid line is $1/T$. Error bars are 95 % confidence bounds to the fits in a).

$Q_\mathrm{b}^{-1}(B) = Q_\mathrm{i}^{-1}(B) - Q_\mathrm{i}^{-1}(B = 0)$. We fit the cESR-spectrum to a model of two coupled oscillators to extract the collective coupling, Ω, and line width γ_2 ($= 1/T_2$ for a Lorentzian cESR peak) of the spin system.

$$S_{21}(\omega) = 1 + \frac{\kappa_c}{i(\omega - \omega_0) - \kappa + \frac{\Omega^2}{i(\omega - \omega_s) - \gamma_2/2}}, \qquad (7)$$

Eq. 7 here describes the central $g = 2$ peak only and $\omega_0 = 2\pi\nu_0$ and $\omega_s = g\mu_\mathrm{B}B/\hbar$ is the angular resonance frequency and induced Zeeman splitting of the spins respectively, and $\kappa_{(c)} = \omega_0/Q_{(c)}$. From the collective coupling Ω we can evaluate the surface spin density based on the geometry of the resonator[6]. Comparing the same resonator before and after annealing also gives a direct measure of the relative reduction in spin density independent of resonator geometry via the observed reduction in collective coupling of the spins, $\Omega \propto \sqrt{n}$. In the 'After' measurement we have removed $\sim 2 \cdot 10^{17}$ Hydrogen spins m^{-2} and the density of $g = 2$ spins was reduced 5.3 times to $\sim 0.17 \cdot 10^{17}$ spins m^{-2}. Supplementary Figure 1e shows the good agreement of the cESR data to theory. We note that the reduction of 5.3 times is larger than previously observed[6], suggesting that the $g = 2$ spins have a larger desorption barrier than the hydrogen.

SUPPLEMENTARY NOTE 6. CW POWER SATURATION MEASUREMENTS: T_1

When evaluating the spin density it is essential to ensure that the spin ensemble is not saturated by the microwave signal in the resonator. To verify this we measure the cESR-spectrum at a wide set of applied powers and extract the dissipation into the spin system at the $g = 2$ peak as a function of circulating power in the resonator. The result for one such measurement (after annealing, evaluated for the $g = 2$ peak) is shown in Supplementary Figure 3a for three different temperatures. The method to evaluate Q_s is described in Ref. [6] together with the methodology used to extract the spin relaxation time T_1 plotted versus temperature in Supplementary Figure 3b. Interestingly we find a T^{-1} dependence of the relaxation time, a signature of direct spin-lattice relaxation as the dominant mechanism for spin energy relaxation [9]. Direct phonon relaxation and a $T_1 \propto T^{-1}$ dependence is also the dominant mechanism for TLS relaxation in amorphous glasses at low temperatures, well captured by both the STM and the GTM [1], predicting a similar dominating phonon relaxation time in the ms range.

We note that spin desorption does not change T_1, while the electron spin dephasing time T_2 inferred from the transition line-width increases marginally (table I), an indication of reduced spin-spin induced decoherence, alternatively the remaining spins could be of a different nature.

SUPPLEMENTARY NOTE 7. NOISE MEASUREMENT SETUP: DUAL POUND LOCKING

The measurement setup we use is a further development of the Pound locking technique [10] for microwave resonators. This modification allows us to simultaneously measure the frequency noise in two different resonators, increasing the amount of data collected and allowing for measurement of correlated noise.

Pound locking is a highly accurate technique to directly measure frequency noise of microwave oscillators. This as opposed to measuring the phase noise S_φ using a homo/heterodyne technique[11]. The advantage is that we gain in sensitivity and the measurement does not suffer from additional complications such as the Leeson effect, and it is especially useful in cryogenic environments, where homo- or heterodyne techniques suffer from a wide range of fluctuations, such as in electrical length in each of the two measurement paths (signal and reference), and thermal fluctuations. The Pound locking technique instead sends the signal and reference through the same physical transmission line, where the reference takes the form of a phase modulated spectrum on top of the signal, making the measurement insensitive to first order in any variations in electrical length. The phase modulation frequency is recovered by a non-linear detector (here

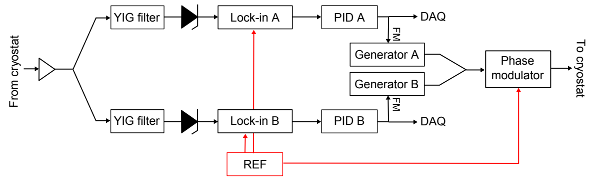

Supplementary Figure 4. Dual Pound-locking measurement setup. For details see text.

a diode) and any deviations in the signal frequency from the resonator frequency causes a beating at the phase modulation frequency. This beating is nulled using a lock-in in series with a PID controller which adjusts the signal frequency sent out by the microwave generator to match the instant resonance frequency.

Instead of a single Pound loop we here run two loops in parallel, as shown in Supplementary Figure 4. Each loop, A and B, works in the same way as described in detail in Ref. [10], locked to the 4.6 and 5.0 GHz resonator respectively. The microwave signals from each loop are combined and sent through the same transmission line in the cryostat, and later selectively split to each arm using 7th order tunable YIG filters with a bandwidth of 40 MHz tuned to each respective resonance frequency. This type of multiplexed setup in principle allows for an arbitrary number of Pound loops in parallel without introducing any cross-coupling and errors in frequency measurement, as long as the YIG filters can selectively isolate the phase modulation spectrum from each measured resonator.

The power applied in each Pound loop was carefully verified using a spectrum analyser and adjusted to be equal in the two measurements, both for power incident on the resonators and power incident on the detector diode.

SUPPLEMENTARY NOTE 8. LOSS TANGENT MEASUREMENTS

To obtain the loss tangent we measure the frequency shift of each resonator while slowly ramping up the temperature of the cryostat over the course of $\sim$ 120 minutes. The frequency is measured using the Pound-loop. The loss tangent $\tan\delta_{\rm i}$ is then extracted from fits of the $\nu_0(T)$ data to the STM (and GTM).

$$\delta\nu(T) = F\tan\delta_{\rm i}\Bigg[\mathrm{Re}\Bigg(\Psi(\frac{1}{2}+\frac{\nu_0 h}{2\pi i k_{\rm B}T}) + \Psi(\frac{1}{2}+\frac{\nu_0 h}{2\pi i k_{\rm B}T_0}) - \ln\frac{T}{T_0}\Bigg)\Bigg]. \quad (8)$$

Here $\delta\nu(T) = (\nu_0 - \nu(T))/\nu_0$, Ψ is the di-gamma function and T_0 is a reference temperature. The measured data and fits to Eq. (8) are shown in Supplementary Figure 5. Extracted parameters are shown in Table 1 in the main manuscript.

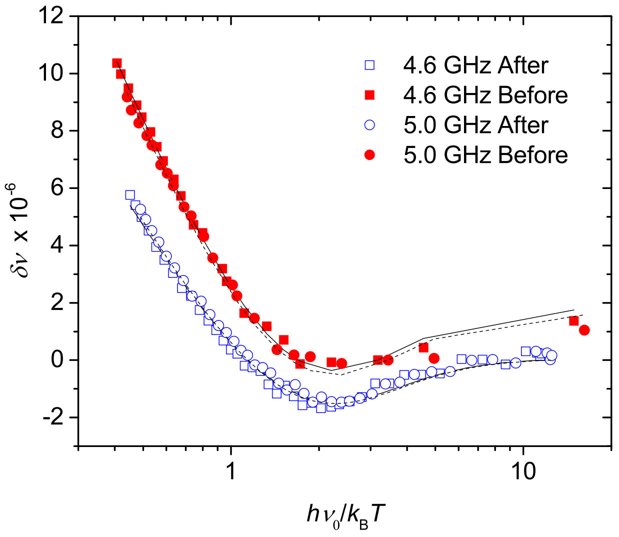

Supplementary Figure 5. Intrinsic loss tangent. Frequency shift of the resonators as a function of temperature and fits to Eq. (8) (black lines). For both resonators we observe a reduction in loss tangent upon surface spin desorbtion by 25-30%. Extracted values for $\tan\delta_\mathrm{i}$ are shown in Table 1. Curves are offset for clarity. Measured using the Pound locking technique.

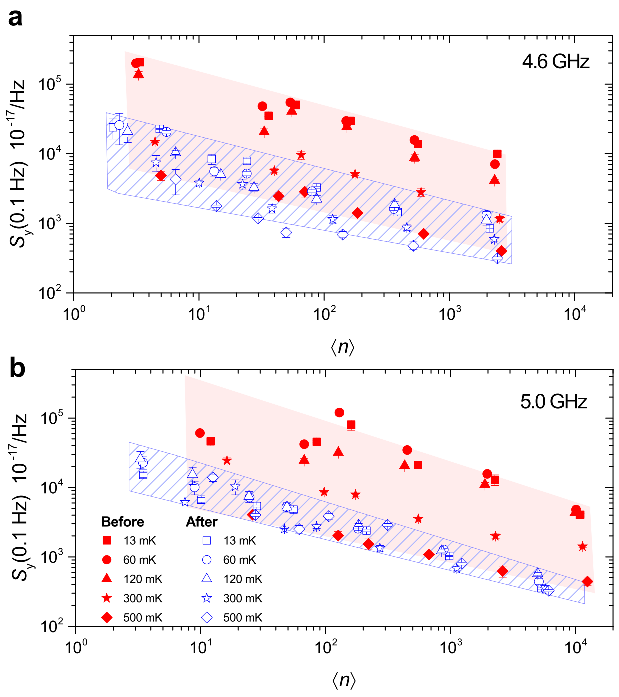

Supplementary Figure 6. Reduction of noise due to spin desorption. Frequency noise power spectral density $S_\mathrm{y}(f) = S_{\delta\nu}(f)/\nu_0^2$ at $f = 0.1$ Hz for the a) $\nu_0 = 4.6$ GHz resonator (same data as in Figure 1c in the main manuscript) and b) the 5.0 GHz resonator. Red solid markers are before, and blue hollow markers are after spin desorption respectively. Shaded regions are a guide for the eye. Error bars indicate the standard deviation of the Allan deviation obtained for a series of time-scales in the $1/f$ noise region (see Supplementary note 9 for details).

SUPPLEMENTARY NOTE 9. NOISE ANALYSIS

The sampled frequency vs time signal recorded from the Pound loop is converted to frequency noise spectral density S_y by calculating the overlapping Allan-variance $\sigma_\mathrm{y}^2(\tau)$ (AVAR) for M discrete samplings $f_k(n\tau)$ at multiples n of the sampling rate τ.

$$\sigma_\mathrm{y}^2(n\tau) = \frac{1}{2(M-1)} \sum_{k=1}^{M-1} (f_{k+1} - f_k)^2 \tag{9}$$

For $1/f$ noise the power spectral density $S_\mathrm{y}(f) = h_{-1}/f$ relates to the Allan variance $\sigma_\mathrm{y}^2 = 2\ln(2)h_{-1}$ via the coefficient $h_{-1} \equiv A_0/2\pi$ [12]. The AVAR is evaluated at several time-scales $t = n\tau$ ranging from 20 to 80 seconds, well within the $1/f$ noise limit, and the average value for h_{-1} is obtained with high statistical significance. Error bars are calculated from the standard deviation of the multiple evaluations of the AVAR in the same time interval. Each datapoint in Supplementary Figure 6 is the result of a 2.8 hours long measurement, collecting 10^5 samples without interruption at a rate $\tau^{-1} = 10$ Hz. Such long measurement times are required to obtain statistically significant results for h_{-1} since the $1/f$ noise in these high-Q resonators is only exceeding the system white noise level at frequencies below $\sim 1 - 0.1$ Hz, in particular at high temperatures and low applied powers and especially in the 'After' measurement where the $1/f$ noise level is significantly lower.

Full data for both resonators measured is shown in Supplementary Figure 6.

SUPPLEMENTARY NOTE 10. TEMPERATURE DEPENDENCE OF S_y

To extract μ we measure the temperature dependence of S_y, which in the low power and low temperature limit is expected to scale as $S_\mathrm{y}(T) \propto A_\mu T^{-(1+2\mu)}$. The low temperature limit is given by $T < h\nu/k_\mathrm{B} \approx 220$ mK for our $\nu = 4.6$ GHz resonator. This measurement and fits to extract μ are shown in Supplementary Figure 7. Confidence intervals given for μ are propagated error bars from the calculation of S_y. Indeed we find $\mu > 0$ for both measurements and whilst error bars are relatively large, we conclude that interaction is still present and μ has not changed by a significant amount. We do not have data at low enough photon numbers to accurately evaluate μ for the 5 GHz resonator.

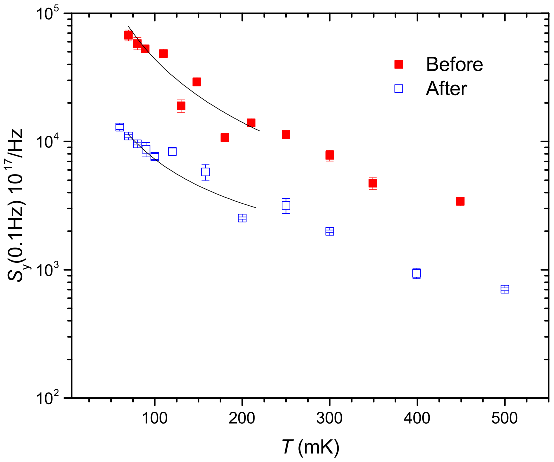

Supplementary Figure 7. Temperature dependence of the noise power spectral density at $\langle N \rangle = 8 \pm 2$ before and $\langle N \rangle = 4 \pm 2$ after surface spin desorption. Solid lines are fits to $S_{\mathrm{y}}(T) = A_{\mu} T^{-(1+2\mu)}$. Before annealing we find $2\mu = 0.64 \pm 0.50$ while after annealing $2\mu = 0.43 \pm 0.21$. Due to thermal saturation, data points below 70 mK are excluded from the fit. We also only consider the low temperature regime $k_{\mathrm{B}}T < \hbar\omega_0 \approx 220$ mK. Error bars indicate the standard deviation of the Allan deviation obtained for a series of time-scales in the $1/f$ noise region (see Supplementary note 9 for details).

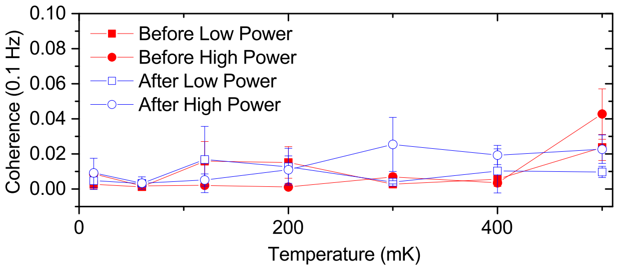

Supplementary Figure 8. Uncorrelated noise. The coherence (normalised correlation) at 0.1 Hz between simultaneously measured 4.6 and 5.0 GHz resonators. Error bars are the standard deviation of the coherence within time scales ±10% from 0.1 Hz. The measurement shows that the $1/f$ noise in each resonator at the time-scale of 0.1 Hz is dominated by local sources at all relevant temperatures. Low power is equivalent to $\langle N \rangle \sim 1 - 10$ and high power corresponds to $\langle N \rangle \sim 10^3 - 10^4$.

SUPPLEMENTARY NOTE 11. CORRELATED NOISE

To rule out external factors, such as system noise, magnetic field or thermal fluctuations, vibrations, and vortices influencing the results we verify that the measured $1/f$ noise is local to each resonator by measuring their correlated noise. We evaluate the correlated noise as the coherence function from the spectral densities

$$C = \frac{|S_{\mathrm{AB}}|^2}{S_{\mathrm{AA}} S_{\mathrm{BB}}}. \tag{10}$$

Here S_{XY} is the cross-power spectral density

$$S_{\mathrm{XY}} = \int_{-\infty}^{\infty} dt e^{-i\omega t} \int_{-\infty}^{\infty} d\tau \nu_{\mathrm{X}}(\tau)\nu_{\mathrm{Y}}(t+\tau) \tag{11}$$

of frequency fluctuations $\nu_{\mathrm{A}}(t)$ and $\nu_{\mathrm{B}}(t)$, where A and B denote the two different resonators. Supplementary Figure 8 shows the measured coherence C(0.1Hz) as a function of temperature and for the two extreme powers applied to each resonator, obtained from the same data as in Supplementary Figure 6. We observe no correlations at the time-scale of 0.1 Hz that is relevant for the $1/f$ noise analysis performed in this work.

As another control experiment we measured the coherence while applying a weak (0.02 mT) external magnetic field perpendicular to the superconducting thin-film plane of the sample at a frequency of 0.2 Hz. The measured coherence is very strong at this particular frequency and its higher harmonics, as shown in Supplementary Figure 9. These measurements clearly verify that we have successfully eliminated any common sources of noise and the dominating contribution to the $1/f$ noise originates

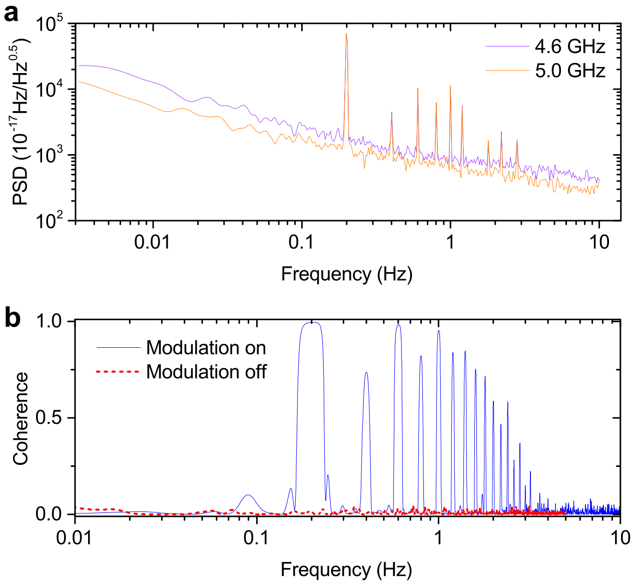

Supplementary Figure 9. Correlated noise due to external perturbation. a) Power spectral density of frequency fluctuations in the two resonators (before spin desorption, at $T = 20$ mK) when a small oscillating magnetic field at 0.2 Hz is applied to the sample. The coherence data for the same measurement as in a) and another measurement when the field modulation is turned off. The amplitude of field fluctuations was 0.1 Oe resulting in approximately 20 kHz oscillations in the resonance frequencies of the resonators.

from noise sources local to each resonator within the entire measurement space presented in this work.

SUPPLEMENTARY REFERENCES

[1] Faoro, L. and Ioffe, L. B. Interacting tunneling model for two-level systems in amorphous materials and its predictions for their dephasing and noise in superconducting microresonators. Phys. Rev. B **91**, 014201 (2015).

[2] Lisenfeld, J. et al. Decoherence spectroscopy with individual two-level tunneling defects. Scientific Reports **6**, 23786 (2016).

[3] Burnett, J., Faoro, L. and Lindström, T. Analysis of high quality superconducting resonators: consequaneces for TLS properties in amorphous oxides. Supercond. Sci. Technol. **29**, 044008 (2016).

[4] de Graaf, S. E., Davidovikj, D., Adamyan, A., Kubatkin, S. E. and Danilov, A. V. Galvanically split superconducting microwave resonators for introducing internal voltage bias. Appl. Phys. Lett. **104**, 052601 (2014).

[5] de Graaf, S. E., Danilov, A. V., Adamyan, A., Bauch, T. and Kubatkin, S. E. Magnetic field resilient fractal superconducting resonators for coupling to free spins. J. Appl. Phys., **112**, 123905 (2012)

[6] de Graaf, S. E., Adamyan, A. A., Lindström, T., Erts, D., Kubatkin, S. E., Tzalenchuk, A. Ya. and Danilov, A. V. Direct identification of dilute surface spins on Al2O3: origin of flux noise in quantum circuits. Phys. Rev. Lett. **118**, 057703 (2017).

[7] Kirsh, N., Svetitsky, E., Burin, A. L., Schechter, M. and Katz, N. Revealing the nonlinear response of a tunneling two-level system ensemble using coupled modes. Phys. Rev. Mat. **1**, 012601 (2017).

[8] Khalil, M. S. et al. Evidence for hydrogen two-level systems in atomic layer deposition oxides. Appl. Phys. Lett. **103**, 162601 (2013).

[9] A. Schweiger, G. Jeschke, Principles of pulse electron paramagnetic resonance, Oxford University Press (New York 2001).

[10] Lindström, T., Burnett, J., Oxborrow, M. and Tzalenchuk, A. Ya. Pound-locking for characterization of superconducting microresonators. Rev. Sci. Instrum. **82**, 104706 (2011).

[11] Barends, R., Hortensius, H. L., Zijlstra, T., Baselmans, J. J. A., Yates, S. J. C., Gao, R. J. and Klapwijk, T. M. Noise in NbTiN, Al, and Ta superconducting resonators on silicon and sapphire substrates. IEEE Trans. Appl. Supercond. **19**, 936-939 (2009).

[12] Rubiola, E. Phase noise and frequency stability in oscillators (Cambridge University Press, 2009).
